# Factors driving persistence to first-line advanced therapies in inflammatory bowel disease: a real-world study from a tertiary referral centre

**DOI:** 10.1007/s11739-025-03943-1

**Published:** 2025-04-25

**Authors:** Marco Vincenzo Lenti, Giovanni Santacroce, Federica Lepore, Francesco Mordà, Antonio Lo Bello, Nicola Aronico, Caterina Mengoli, Mariangela Delliponti, Raphael Frondana, Iara Moreira Frondana, Antonio Di Sabatino

**Affiliations:** 1 Department of Internal Medicine and Medical Therapeutics, University of Pavia, Pavia, Italy; 2 First Department of Internal Medicine, Clinica Medica I, Fondazione IRCCS Policlinico San Matteo, Università Di Pavia, Piazzale Golgi 19, 27100 Pavia, Italy; 3 SSD Biostatistica and Clinical Trial, Fondazione IRCCS Policlinico San Matteo, Pavia, Italy; 4 Universidade de São Paulo, São Paulo, Brazil

**Keywords:** Biologics, Crohn’s disease, Therapy discontinuation, Oral small molecules

## Abstract

Factors driving the persistence of advanced therapies—defined as the duration from therapy initiation to discontinuation—in inflammatory bowel disease (IBD) remain unclear. This study aimed to evaluate the persistence of biologics and oral small molecules in a real-word IBD cohort and to identify influencing factors. Data from IBD patients starting advanced therapy at a tertiary referral centre after 2010 were retrospectively collected, including persistence and discontinuation reasons. Differences in persistence probability among therapies were analysed, and factors influencing persistence versus discontinuation due to failure were assessed by univariate and multivariate analyses. Among 274 included patients [median age 42.5 years; F/M 119/155; 146 with Crohn’s disease (CD) and 128 with ulcerative colitis; median follow-up 38 months (IQR 14–75)], 141 (51.5%) remained persistent with first-line therapy, while 70 (26%) discontinued due to failure. No significant difference in persistence was observed among drugs (*p* = 0.11). Univariate analysis identified CD phenotype (*p* < 0.01), disease duration prior to therapy (*p* = 0.01), concomitant mesalamine or steroids (*p* < 0.01), and therapy optimisation (*p* < 0.01) as factors influencing persistence. Multivariate analysis confirmed CD phenotype as associated with higher persistence, while therapy optimisation was linked to increased discontinuation risk. CD was associated with better drug persistence, while therapy optimisation correlated with a higher discontinuation rate. Targeting deep healing and enhancing timely, precise optimisation strategies is essential for improving treatment outcomes.

**Graphical abstract:**

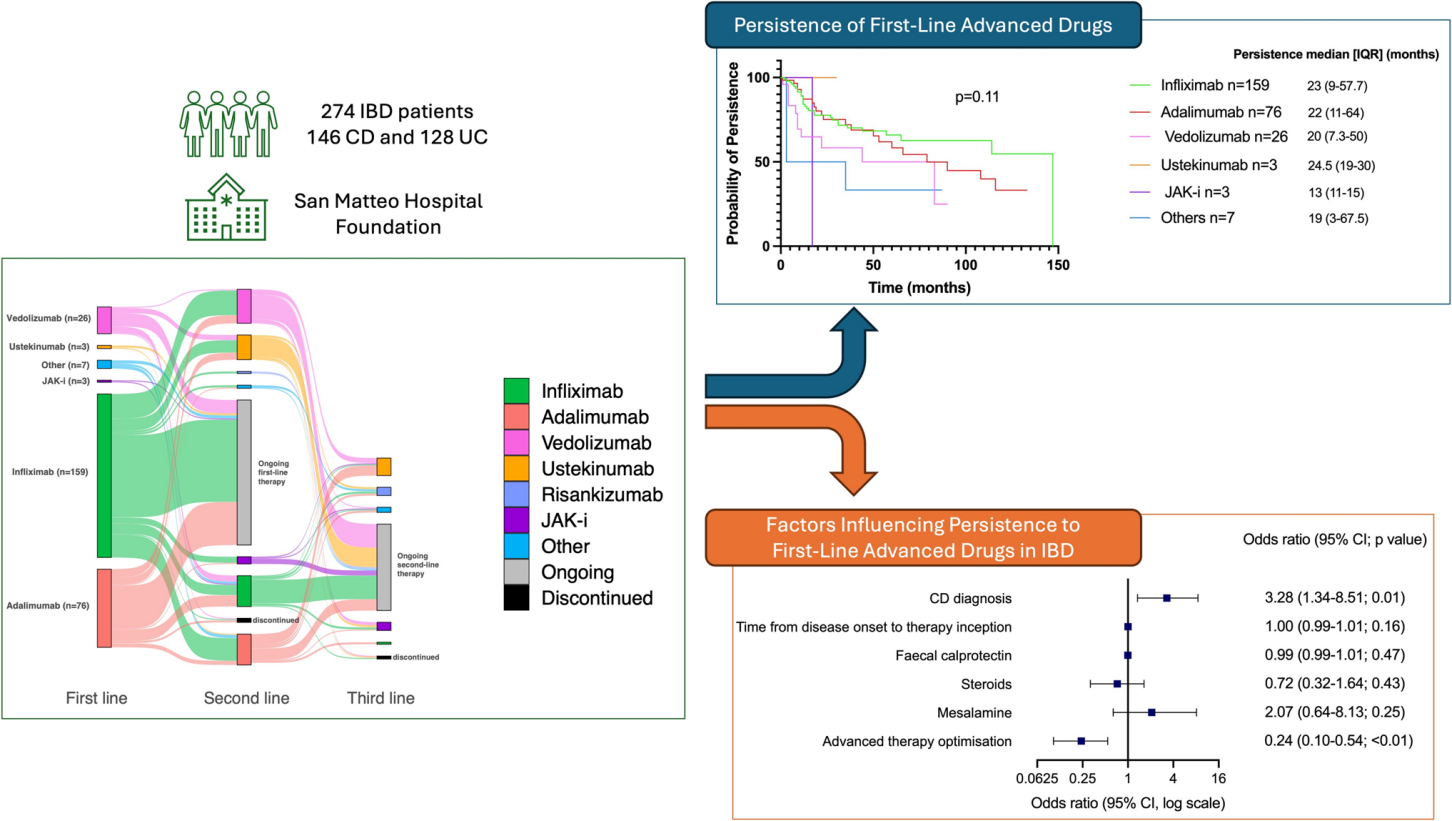

## Introduction

Several advanced therapies, including biologics and oral small molecules, are available for the treatment of inflammatory bowel disease (IBD), a chronic immune-mediated disorder of the gastrointestinal tract [1, 2]. Despite the growing range of therapeutic options, remission rates in IBD remain modest, with a “therapeutic ceiling” of approximately 30% [3, 4]. Physician managing IBD patients often encounter significant challenges, including primary non-response, secondary loss of response, adverse events, and poor tolerance to prescribed medications, which complicates therapeutic decision-making and patient management [5].

In this challenging scenario, the concept of persistence to therapy—i.e. the duration from initiation to its discontinuation—has recently emerged as a valuable real-word indicator of drug efficacy and tolerability [6, 7]. A recent meta-analysis compared persistence across advanced therapies in IBD, revealing superior persistence with Ustekinumab and Vedolizumab compared to anti-tumour necrosis factor (TNF) agents [8]. However, a crucial gap remains in understanding the factors driving therapy persistence or discontinuation in a real-world setting. Identifying these factors is key to optimising and personalising treatment strategies in IBD, reducing the therapeutic failure rates, improving patient outcomes, and ultimately lowering healthcare costs.

This study aims to assess the persistence of first-line advanced therapies in a real-world setting at a tertiary Italian IBD referral centre and to identify factors influencing treatment persistence versus discontinuation in IBD patients.

## Materials and methods

### Study population and design

This observational study was conducted at a tertiary IBD referral centre, i.e. San Matteo Hospital Foundation, Pavia, Italy. This study is part of a larger project (“ON Foods-Research and innovation network on food and nutrition Sustainability, Safety and Security–Working ON Foods”) focussing on malnutrition and other factors in relation to response to drugs in IBD, addressing a secondary ancillary aim of the research.

469 adult patients (≥ 18 years) with an established diagnosis of Crohn’s disease (CD) and ulcerative colitis (UC), as per the European Crohn’s and Colitis Organisation (ECCO) guidelines [9], who initiated first-line advanced therapies after the year 2010 were prospectively enrolled. According to the ECCO guidelines, patients initiating advanced therapy due to moderate-to-severe disease activity and not responding to conventional therapy or steroid dependant were included [10, 11]. In addition, therapy initiation was considered for patients with upper gastrointestinal involvement, postsurgical prophylaxis, or perianal disease. Therapeutic drug monitoring (TDM) was not applied.

Only 274 patients who had their last follow-up visit or were reachable by telephone between May and September 2024 were retrospectively selected to ensure a reliable and updated assessment of the exact timing of potential treatment discontinuation or long-term persistence. Demographic and clinical data were collected.

The primary aim of the study was to assess persistence versus discontinuation of first-line advanced therapies in a real-world setting. Other aims of the study were to identify potential sociodemographic or clinical factors influencing treatment persistence and to evaluate differences in persistence across various first-line advanced therapies.

The main study endpoint, namely persistence as a surrogate for drug efficacy, was analysed by comparing patients who persisted with treatment to those who discontinued due to treatment failure.

### Data collection

#### Socio-demographic data

Collected baseline sociodemographic data included sex, age at diagnosis (years), body mass index (BMI), family history of IBD, smoking status (active smoker, non-smoker, or ex-smokers if quit for over 5 years), education level (higher education defined as a minimum of 9 years of formal schooling), household income (low income defined as < 1000 euro/month), and marital status (single/living alone or living with a partner).

#### Clinica data

Clinical data at therapy inception included IBD phenotype (CD or UC), age at IBD diagnosis (years), comorbidities (assessed by the Charlson Comorbidity Index) [12], extra-intestinal manifestations (i.e. arthritis/arthralgias, ocular manifestations, erythema nodosum, pyoderma gangrenosum, aphthous stomatitis, and primary sclerosing cholangitis), IBD-related complications (including fistulae, abscesses, bowel obstruction, cancer, and toxic megacolon) and surgery history. Laboratory data included C-reactive protein (CRP) and faecal calprotectin. Clinical disease activity was assessed using the Harvey–Bradshaw Index (HBI) for CD and the partial Mayo (pMayo) score for UC: remission was defined as HBI < 5 or pMayo 0–1; mild disease as HBI 5–7 or pMayo 2–4; moderate disease as HBI 8–16 or pMayo 5–6; severe disease as HBI > 16 or pMayo 7–9. Endoscopic activity was evaluated by the Simple Endoscopic Score for CD (SES-CD) and Mayo Endoscopic Score (MES) for UC: remission was defined as SES-CD 0–2 or MES 0; mild activity as SES-CD 3–6 or MES 1; moderate activity as SES-CD 7–15 or MES 2; severe disease as SES-CD > 15 or MES 3.

#### Medication and persistence data

Data on first-line advanced therapy included time from disease onset to therapy initiation, type of drug (i.e. biologics or small oral molecules), route of administration, need for therapy optimisation (including adjusting administration frequency or increasing the drug dose, as per regulatory guidelines for each medication), and concomitant use of mesalamine, steroids, or immunosuppressants (initiated simultaneously with or within 3 months of starting advanced therapy). Information on second-line and third-line advanced medications were also collected, when applicable.

Total duration of first-line therapy was recorded, with patients still on medication as of September 2024 considered persistent. Reasons for discontinuation were documented, including failure (i.e. primary or secondary non-response), allergy/intolerance, long-term (at least > 12 months) remission, immunogenicity, or other/unknown causes.

### Statistical methods

Data were analysed using GraphPad Prism v10.3.1 (GraphPad Software, Boston, Massachusetts, USA). Continuous data were summarised as medians and interquartile ranges (IQRs), while categorical data were presented as counts and percentages. Missing data were excluded from statistical calculations. A *p* value < 0.05 was considered as statistically significant.

Kaplan–Meier survival analysis and the log-rank test were used to compare persistence probability across therapies.

Univariate analyses were conducted to assess differences between persistence and discontinuation due to failure of first-line therapy, with Fisher’s exact test for categorical variables and t-test or Mann–Whitney for continuous variables. Discontinuation for reasons other than failure was not included in this analysis. The normality of distributions was assessed using the Shapiro–Wilk test. Multivariate analysis was performed using logistic regression models, with odds ratios reported as exponential of *β*-values. The Hosmer–Lemeshow test assessed model goodness-of-fit.

### Ethical approval

The study was approved by the San Matteo Hospital Foundation Ethics Committee (Protocol No. 17919, 2024, dated March 19, 2024) and adhered to the ethical guidelines of the 1975 Declaration of Helsinki (6 th revision, 2008). Informed consent was obtained from all the participants. Members of the public were not involved into the design of this research project. Raw data cannot be made public due to privacy restriction but can be shared upon reasonable request to the corresponding author. The STROBE guideline was followed for quality assurance.

## Results

### Population characteristics

274 IBD patients were retrospectively included in the study, with a median age of 42.5 years (IQR 29–56.2), F/M 119/155. 146 patients had CD and 128 had ulcerative colitis. Baseline demographics and clinical features of the study population are detailed in Table 1.

**Table 1:** Persistence vs discontinuation due to failure of first-line advanced therapy in IBD patients

		Total	Persistence	Discontinuation due to failure	*p*^§^
Patients		274 (100)	141 (51.5)	70 (25.5)	–
Socio-demographic features at first-line therapy inception					
Sex	Female *n*. (%)	119 (43)	55 (39)	32 (46)	0.35
	Male *n*. (%)	155 (57)	86 (61)	38 (54)	
Age	median [IQR] (years)	42.5 [29–56.2]	37 [23–53]	40 [28–50]	0.77
BMI	median [IQR] (Kg/m^2^)	24 [20.8–26.7]	24 [20.5–26.7]	24.9 [22.9–28]	0.14
Family history of IBD	*n*. (%)	20 (10)	12 (11)	5 (11)	0.90
Level of education	> 9 years *n*. (%)	127 (81)	68 (77)	32 (80)	0.73
Household income	> 1000 euro/month *n*. (%)	115 (85)	65 (88)	30 (79)	0.21
Marital status	Lives alone/single *n*. (%)	86 (54)	48 (54)	23 (56)	0.82
	With a partner *n*. (%)	72 (46)	41 (46)	18 (44)	
Smoking habit	No *n*. (%)	137 (53)	70 (52)	36 (55)	0.12
	Active smoker	49 (19)	29 (22)	7 (11)	
	Ex-smoker	72 (28)	35 (26)	23 (35)	
Clinical features at first-line therapy inception					
IBD phenotype	CD	146 (53)	79 (56)	23 (33)	** < 0.01**
	UC	128 (47)	62 (44)	47 (67)	
Age at diagnosis	median [IQR] (years)	28 [20–41]	28.5 [21–40]	32.5 [20.7–43.3]	0.54
Charlson comorbidity index > 1	*n*. (%)	64 (26)	27 (19)	19 (28)	0.17
Extra-intestinal manifestations	*n*. (%)	91 (33)	42 (30)	25 (36)	0.40
Complications	*n*. (%)	52 (19)	22 (16)	8 (11)	0.39
Need for surgery	*n*. (%)	61 (22)	27 (19)	14 (20)	0.88
Faecal calprotectin	median [IQR] (mg/Kg)	500 [150–1607]	385 [145–1390]	550 [250–2100]	0.09
CRP	median [IQR] (mg/dL)	0.7 [0.2–2.5]	0.66 [0.2–2]	0.54 [0.1–5.1]	0.96
Clinical activity^#^	Remission *n*. (%)	18 (8)	13 (11)	3 (5)	0.16
	Mild *n*. (%)	77 (35)	42 (35)	17 (31)	
	Moderate *n*. (%)	85 (39)	44 (37)	18 (33)	
	Severe *n*. (%)	39 (18)	20 (17)	17 (31)	
Endoscopic activity^°^	Remission *n*. (%)	13 (7)	6 (6)	3 (5)	0.27
	Mild *n*. (%)	42 (22)	27 (28)	13 (24)	
	Moderate *n*. (%)	96 (50)	46 (47)	21 (32)	
	Severe *n*. (%)	42 (22)	18 (19)	18 (28)	
CD location (Montreal classification)	L1 (ileal)	26 (18)	23 (29)	3 (13)	0.07
	L2 (colonic)	8 (5)	4 (5)	4 (17)	
	L3 (ileocolonic)	66 (45)	51 (65)	15 (65)	
	L4 (upper GI) modifier	5 (3)	4 (5)	1 (4)	
UC location (Montreal classification)	Proctitis n. (%)	7 (5)	7 (11)	0	**0.04**
	Left-side colitis n. (%)	35 (27)	17 (27)	18 (38)	
	Pancolitis n. (%)	66 (52)	37 (60)	29 (62)	
First-line therapy					
Time from disease onset to advanced therapy inception	median [IQR] (months)	21 [9–56]	25 [10–61]	45 [15.5–102]	**0.01**
Calendar years	2010–2017 *n*. (%)	80 (29)	25 (18)	19 (27)	0.15
	2018–2024 *n*. (%)	194 (71)	116 (82)	51 (72)	
Advanced therapy	Infliximab *n*. (%)	159 (58)	80 (57)	33 (47)	0.25
	Adalimumab *n*. (%)	76 (28)	42 (30)	21 (30)	
	Vedolizumab *n*. (%)	26 (9.5)	13 (9)	11 (16)	
	Ustekinumab *n*. (%)	3 (1)	2 (1)	0	
	JAK-i *n*. (%)	3 (1)	2 (1)	1 (1)	
	Others *n*. (%)	7 (2.5)	2 (1)	4 (6)	
Drug RoA	IV	150 (55)	74 (52)	43 (61)	0.11
	SC	113 (42)	64 (45)	23 (33)	
	P.O	8 (3)	3 (2)	4 (6)	
Therapy optimisation	*n*. (%)	69 (28)	29 (21)	32 (50)	** < 0.01**
Mesalamine	*n*. (%)	195 (80)	102 (74)	57 (90)	** < 0.01**
Steroids	*n*. (%)	112 (45)	54 (39)	38 (60)	** < 0.01**
Immunosuppressant	*n*. (%)	48 (19)	27 (20)	11 (17)	0.65

*BMI* body mass index, *CD* Crohn’s disease, *CRP* C-reactive protein, *IBD* inflammatory bowel disease, *IQR* interquartile range, *IV* intravenous, *JAK-i* Janus-kinase inhibitor, *P.O*. by mouth, *RoA* route of administration, *SC* subcutaneous, *UC* ulcerative colitis

^§^Univariate analyses compared persistence vs discontinuation groups

^*^not mutually exclusive

^#^assessed by HBI for CD and pMayo for UC

°assessed by SES-CD for CD and Mayo endoscopic score for UC. Missing data were excluded from percentage calculation

Significant *p*-values are in bold

**Fig. 1:**
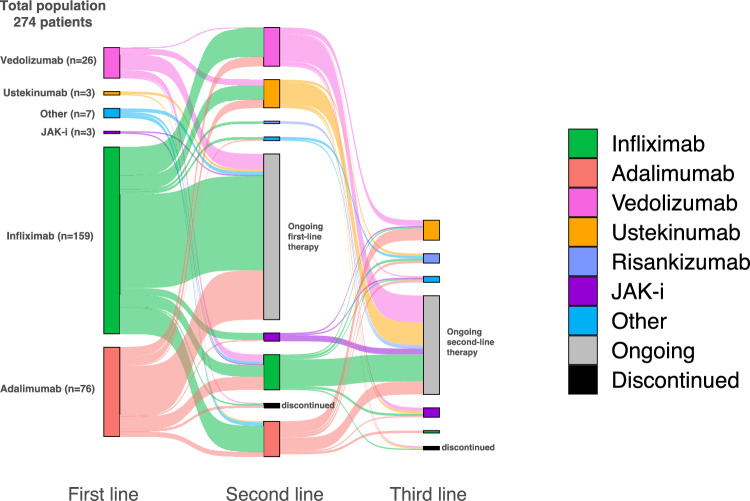
Progression of advanced drug therapies across first-, second-, and third-line treatments. This Sankey diagram illustrates the progression through successive lines of treatment (biologics and oral small molecules) in our cohort of 274 IBD patients. Starting from their first-line medication (left side of the diagram), patients may continue with the same medication, discontinue, or switching to other drugs. The diagram depicts transitions from first-line to second- and third-line therapies

In our cohort of 274 IBD patients, 159 (58%) started Infliximab, 76 (27.7%) Adalimumab, 26 (9.5%) Vedolizumab, three (1%) Ustekinumab, three (1%) Janus-kinase inhibitors (JAK-i), and seven (2.5%) other advanced therapies as first-line treatments (Fig. 1). The reasons for starting first-line advanced therapy were as follows: 239 (87%) patients for active disease, including those unresponsive to conventional therapy or steroid dependant; 16 (5.8%) for postsurgical prophylaxis; 14 (5%) for active isolated perianal disease; 5 (1.8%) for active isolated upper GI involvement.

Among the 274 patients enrolled, 129 (47%) switched to a second-line therapy, and 40 (14.6%) subsequently to a third-line therapy. Of the 129 patients switching to a second-line therapy, 30 patients (23.2%) started Infliximab, 30 (23.3%) Adalimumab, 33 (25.6%) Vedolizumab, 24 (18.6%) Ustekinumab, two (1.6%) Risankizumab, seven (5.4%) JAK-i and three (2.3%) other advanced therapies. Of the 40 patients who proceeded to a third-line therapy, two patients (5%) switched to Infliximab, 17 (43%) to Ustekinumab, eight (20%) to Risankizumab, eight (20%) to JAK-i, and five (12.5%) to other advanced therapies.

The median follow-up (from therapy initiation to last 2024 follow-up) was 38 months (IQR 14–75.75).

### Persistence of first-line therapy

**Fig. 2:**
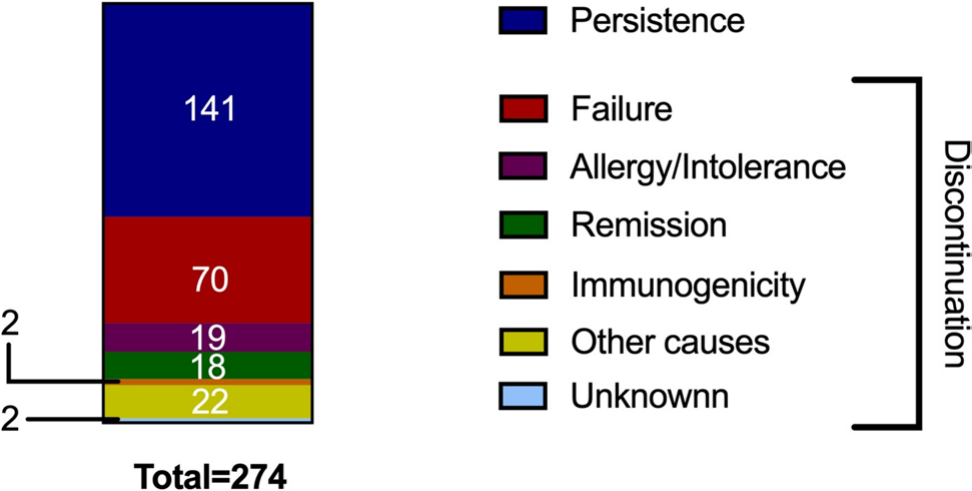
Persistence vs. discontinuation of first-line advanced drugs. This bar plot graphically summarises the rates of persistence and discontinuation in our study population. Different causes of discontinuation are reported

Persistence of first-line therapy was observed in 141 patients (51.5%). Discontinuation occurred in 70 patients (26%) due to treatment failure, 19 (6.9%) due to allergy/intolerance, 18 (6.6%) due to remission, two (0.7%) due to immunogenicity, and 24 (8.8%) for other/unknown reasons (Fig. 2).

**Fig. 3:**
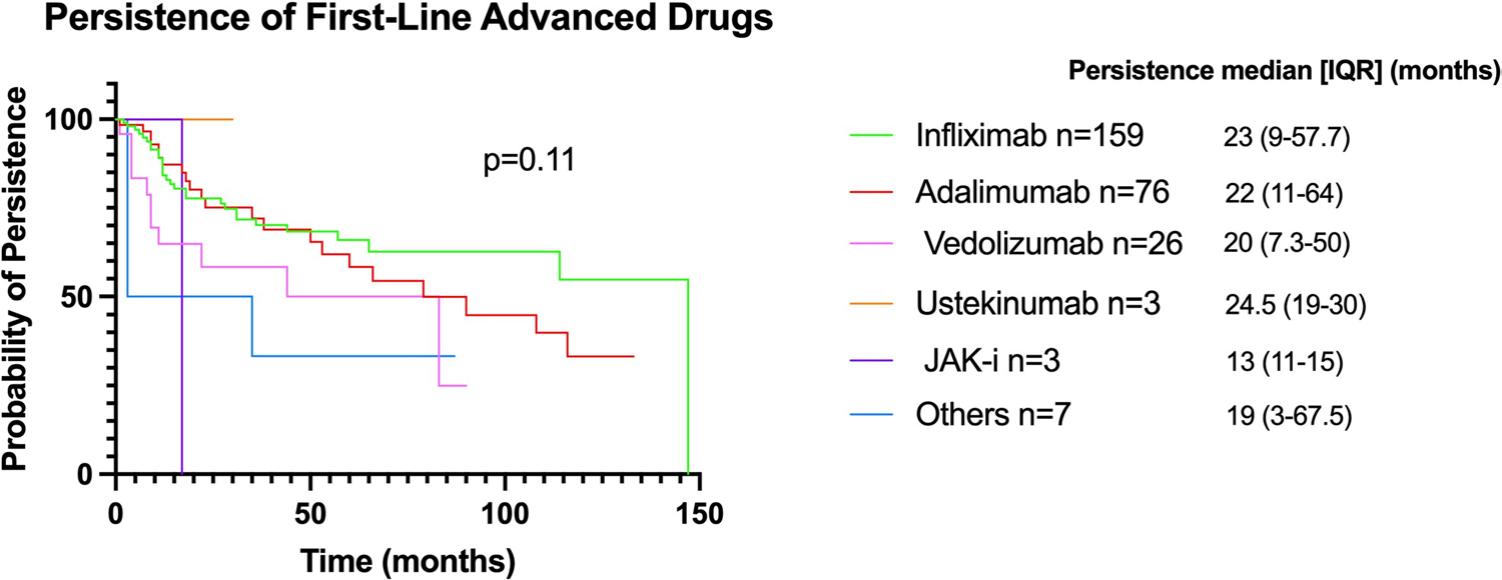
Persistence probability among different first-line advanced drugs. This Kaplan–Meier plot illustrates the persistence probability across various first-line medications, with no statistically significant difference (log-rank *p* value = 0.106). Median persistence across various therapies and interquartile ranges are shown on the right side. *IQR* interquartile range, *JAK-I* Janus-kinase inhibitors

The overall median medication persistence duration was 21 months (IQR 9–56), with drug-specific persistence as follows: Infliximab 23 months (IQR 9–57.5), Adalimumab 22 months (IQR 11–64), Vedolizumab 20 months (IQR 7.25–50), Ustekinumab 24.5 months (IQR 19–30), JAK-i 13 months (IQR 11–15), and other medications 19 months (IQR 3–67.5). No significant difference in persistence probability was observed among the different drugs (*p* = 0.11; Fig. 3).

### Factors influencing persistence versus discontinuation due to failure of first-line advanced therapy

#### Univariate analysis

Univariate analyses identified factors distinguishing patients who persisted on a first-line therapy (141/211, 66.8%) from those who discontinued due to treatment failure (70/211, 33.2%), as shown in Table 1. Patients with CD showed higher persistence rates (56% vs 33%), while UC patients were more likely to discontinue (67% vs 44%) (*p* < 0.01). Patients in the discontinuation group had a significantly longer disease duration compared to the persistence group (45 vs 25 months, *p* = 0.01). When differentiating the disease duration according to the disease phenotype, CD patients showed a non-significantly shorter disease duration compared to UC: 25 months (IQR 6–132) vs 53.5 (IQR 20–111); *p* = 0.06. No significant difference was seen according to the disease location according to the Montreal classification in CD patients (*p* = 0.07), while proctitis was more frequent in UC patients persistent to first-line therapy (*p* = 0.04).

Moreover, therapy optimisation was more frequent among patients who discontinued (50% vs 21%, *p* < 0.01), as was the concomitant use of mesalamine or steroids (90% vs 74% and 60% vs 39%, respectively; *p* < 0.01).

Consistently with the survival analysis, no significant difference was observed between different therapies, and although subcutaneous administration was more common in the persistence group (45%, vs 33%), this difference was not statistically significant (*p* = 0.11).

A trend towards higher faecal calprotectin levels at baseline was observed in the discontinuation group (550 vs 385 mg/kg, *p* = 0.09), while no significant difference in clinical or endoscopic activity was identified between the groups.

Given the evolution of therapy indications and choices over the 15-year period considered, an analysis based on calendar years was conducted. Patients who initiated treatment between 2010 and 2017 were compared to those who started from 2018 onward, revealing no significant differences between the persistence and the discontinuation groups.

#### Multivariate analysis

**Fig. 4:**
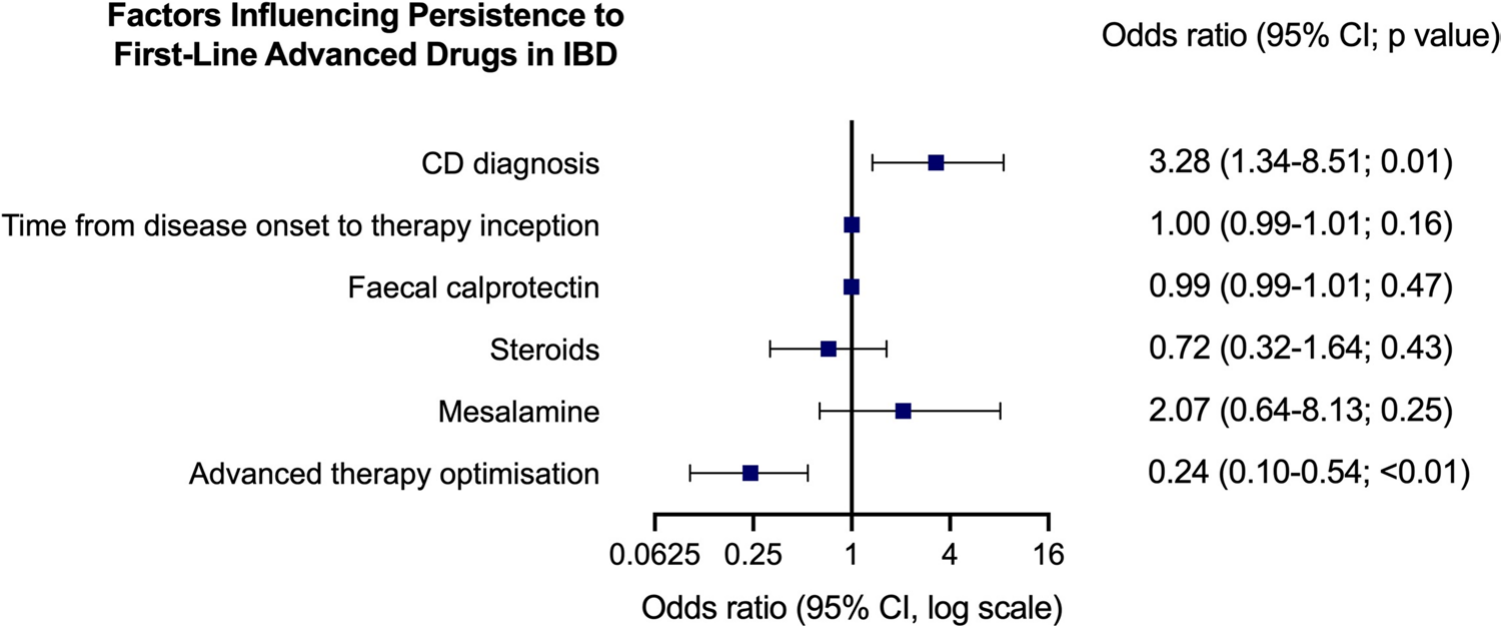
Multivariate analysis of factors associated with persistence of first-line advanced drugs. This forest plot presents the results of multivariate analysis assessing factors associated with persistence to advanced drugs. Odds ratios, 95% confidence intervals, and *p* values are shown on the right side. CD phenotype is associated with higher persistence, while therapy optimisation resulted a risk factor for discontinuation. *CD* Crohn’s disease, *CI* confidence interval, *IBD* inflammatory bowel disease

Multivariate analysis (Fig. 4) confirmed that CD phenotype was associated with higher persistence, with an odds ratio of 3.285 (95% CI 1.344–8.517, *p* = 0.01). Conversely, therapy optimisation was identified as a risk factor for discontinuation, with an odds ratio of 0.24 (95% CI 0.10–0.54, *p* < 0.01). The other variables fitted into the model did not reach statistical significance. The multivariate model showed a good fit (Hosmer–Lemeshow *p* = 0.55).

## Discussion

Our retrospective study addresses a key aspect of the management of patients with IBD on advanced therapies. We observed that slightly more than half of our patients (141/274) continued their medications, while the remnants discontinued, most often due to treatment failure. We found no significant difference in persistence rates across therapies. In addition, we identified multiple factors associated with persistence versus discontinuation due to failure, with CD phenotype and therapy optimisation emerging as key factors influencing persistence at multivariate analysis.

The optimal selection of first-line therapy, as well as the subsequent drug positioning, remains a topic of debate [13]. In our real-world cohort, anti-TNF therapies were the preferred first-line therapy, while Vedolizumab and Ustekinumab were used as first-line in a small percentage of cases. This approach aligns with the available evidence, showing that anti-TNF drugs should be the preferred first-line therapy [14–16]. In terms of second- and third-line therapies, Vedolizumab and Ustekinumab emerged as the preferred second-line options in our cohort, followed by Risankizumab and JAK-i as third-line treatments, in line with available literature [14, 15, 17]. While the first head-to-head trials have begun assessing the efficacy of different drugs in achieving therapeutic targets in CD and UC [18–20], further studies are eagerly awaited to refine therapeutic decision-making.

Similarly to other real-world evidence [21], our study found that approximately 25% of patients discontinued first-line therapy due to treatment failure, leading to unfavourable patient outcomes, increased healthcare costs, and the additional challenge of higher non-response rates with subsequent treatment lines [22]. However, no biomarker currently exists that can reliably guide the selection of the most effective therapy for IBD [23]. Identifying factors that influence treatment persistency could be highly valuable for physicians.

To this end, we first evaluated whether different medications had an impact on treatment persistence. We did not observe differences in persistence probability among advanced therapies, with a median persistence duration of 21 months. While a recent meta-analysis reported superior persistence for Ustekinumab and Vedolizumab over anti-TNF agents [8], the high heterogeneity, primarily due to the inclusion of bio-experienced patients, may have influenced the result. In contrast, our study specifically assessed persistence to first-line therapy, providing a more targeted assessment. Consistently with previous studies [24], subcutaneous administration showed a slightly higher persistence rate compared with the intravenous administration, though this difference was not statistically significant.

CD was associated with greater treatment persistence compared to UC. IBD organisations advocate for a deeper remission target in CD, i.e. transmural healing, which is linked to better outcomes [25]. Therefore, it is likely that advanced therapies are introduced earlier in the natural history of CD compared to UC. Indeed, in our study the disease duration before starting a first-line medication was shorter in the CD cohort compared to the UC one. It remains key for clinicians to apply a treat-to-target strategy aimed at achieving stable, deep and quick remission in both CD and UC, in order to improve treatment outcomes and support cost-effective care. In addition, promoting adherence is paramount, as CD patients have shown better adherence to advanced therapies [26], also due to a reluctance of this cohort in undergoing surgery.

Therapy optimisation was associated with an increased risk of discontinuation in both univariate and multivariate analyses. This underscores the importance of close patient follow-up during advanced therapy to accurately assess treatment efficacy, enabling informed and timely therapeutic decisions. Recently, TDM has emerged as a valuable tool in this process, offering a reliable tool for guiding real-time therapeutic management and improving treatment persistence [27, 28].

Other factors, despite not significant at multivariate analysis, showed a potential role in influencing treatment persistence. A longer disease duration correlated with higher rates of drug discontinuation, suggesting the importance of timely therapy initiation to reduce therapeutic failure in IBD [29, 30]. Moreover, the concurrent initiation of mesalamine or steroids with advanced therapy was associated with higher discontinuation risk. These drugs, which do not have any additional benefit on advanced therapy response [31, 32], can delay prompt therapeutic monitoring and management, increasing the risk of failure and discontinuation.

Clinical and endoscopic inflammatory activity at baseline did not show any impact on treatment persistence. Although a trend towards higher baseline levels of faecal calprotectin was found in the discontinuation group, this was not confirmed in the multivariate analysis. These results suggest that the baseline degree of inflammation does not influence drug persistence, while we can hypothesise that drug response is more likely driven by the specific molecular inflammatory pathways involved [33].

Our study has some limitations that should be mentioned. First, as a retrospective study, it is subject to biases related to data retrieval and its relatively small sample size did not allow to control other variables. The single-centre nature may introduce selection bias due to the specific patient population. Larger, multicentre prospective studies are needed to confirm and expand our findings, with a broader representation of drugs beyond anti-TNF and vedolizumab, which account for only a small subset of our first-line cohort. A larger sample size would also be highly valuable for evaluating persistence and factors affecting it in second- and subsequent lines of therapy.

Despite its limitations, our study provides interesting findings, highlighting the importance of achieving deep therapeutic targets in IBD, fostering patient adherence, and advancing molecular phenotyping efforts for appropriate and personalised decision-making [34]. In addition, it brings attention to the potential role of TDM in enabling swift and appropriate therapy optimisation to prevent increased discontinuation rates and treatment failure. While evidence remains limited, our results suggest the importance of timely therapy initiation and appropriate therapy optimisation or switching, which should not be unduly influenced by concomitant symptomatic medications.

## Data Availability

All relevant data pertaining to the study have been reported here; additional raw data can be shared upon reasonable request to the corresponding author.

## Abbreviations

AI: Artificial intelligence
BMI: Body mass index
CD: Crohn’s disease
CRP: C-reactive protein
ECCO: European crohn’s and colitis organisation
HBI: Harvey–bradshaw index
IBD: Inflammatory bowel disease
IQR: Interquartile range
JAK-i: Janus-kinase inhibitors
MES: Mayo endoscopic score
pMayo: Partial mayo score
SES-CD: Simple endoscopic score for crohn’s disease
TDM: Therapeutic drug monitoring
TNF: Tumour necrosis factor
UC: Ulcerative colitis
